# Parental care reduces parasite-induced mortality in a coral reef fish

**DOI:** 10.1098/rspb.2024.1966

**Published:** 2024-10-16

**Authors:** Alexandra S. Grutter, Simone P. Blomberg, Berilin Duong, Bronwyn E. Fargher, William E. Feeney, Mark I. McCormick, Matthew D. Nicholson, Paul C. Sikkel, Robert R. Warner, Armand M. Kuris

**Affiliations:** ^1^ School of the Environment,The University of Queensland, St Lucia, QLD 4072, Australia; ^2^ Doñana Biological Station, Spanish National Research Council (CSIC), Seville, Spain; ^3^ Coastal Marine Field Station, School of Science, University of Waikato, Tauranga 110, New Zealand; ^4^ Department of Marine Biology and Ecology, Rosenstiel School of Marine, Atmospheric Science, and Earth Science, University of Miami, Coral Gables, FL 33124, USA; ^5^ Water Research Group, Unit of Environmental Sciences, North-West University, Potchefstroom 2520, South Africa; ^6^ Department of Ecology, Evolution, and Marine Biology, University of California, Santa Barbara, CA 93106, USA; ^7^ Marine Science Institute, University of California, Santa Barbara, CA 93106, USA

**Keywords:** parasitism, parental care, Gnathiidae, isopod parasites, parasites, fish defensive behaviour

## Abstract

Settlement patterns of juvenile fish shape coral reef communities. During the recruitment process, predation rates are extremely high. However, the role that parental care plays in reducing mortality, especially by cryptic natural enemies such as parasites, remains largely unstudied. We investigated whether parental care in the spiny chromis damselfish (*Acanthochromis polyacanthus*) protects juveniles from parasite-induced mortality by gnathiid isopods (*Gnathia aureamaculosa*). Using laboratory experiments, we found that survival of recently hatched juveniles when exposed to gnathiids was higher when parents were present (77%) than when parents were absent (25%). Investigation of their faeces in the field and laboratory indicates that adults consume gnathiids. Together, our data suggest that parental care plays a key role in reducing parasite-induced mortality of juvenile spiny chromis via parental consumption of gnathiids. This highlights the overlooked role of parasites as a source of high mortality in juvenile coral reef fishes and the composition of coral reef fish communities.

## 1. Introduction

Most coral reef fishes exhibit a bipartite life-history consisting of a pelagic larval phase, followed by the reef-associated juvenile and adult stages [1]. As juvenile fishes recruit to the reef, predation rates are extremely high, and size-selective, resulting in a ‘predation gauntlet’ during settlement [2,3]. This bottleneck has been used to help understand how predator–prey interactions shape community composition, as well as to explore the evolution of defensive traits in juvenile fishes [4–6]. For example, since high predation rates on juvenile fishes decline rapidly with increasing fish size, predators can dramatically change the biodiversity and abundance of fish communities [4]. Additionally, by influencing the relative abundance of their fish prey, piscivores can also structure fish communities [6]. Finally, size-biased predation can select for mutualisms between fishes and anemones [5]. However, while the role that predation from piscivores plays in shaping recruitment patterns is well recognized, the role that more cryptic enemies, such as parasites, play in shaping patterns of fish mortality remains poorly understood.

Predators and parasites as enemies of prey and hosts comprise a broad array of consumer–prey interactions [7]. The threat posed by an enemy can change according to the victim’s life stage or body condition, which can have important consequences for the outcome of an interaction [8]. For instance, gnathiid isopods (Gnathiidae) are the most common fish ectoparasites across the world’s coral reefs [9]. During their life cycle, gnathiids exhibit three juvenile stages and one adult stage, with most of their life cycle spent as free-living organisms in the benthos, except for stages feeding on fish, which on teleosts can be as brief as a few hours [9]. Gnathiid juveniles feed on the blood of one fish during each stage, and the pathology of gnathiids includes effects on host physiology (e.g. reduced haematocrit, growth, and condition, increased corticosteroids), behaviour (e.g. increased cleaning behaviour), performance (e.g. reduced cognitive performance) and survival (e.g. reduced in gnathiid ‘super-infections’) [9]. After feeding they become visibly engorged with blood and detach from the host, indicating they had completed feeding on the host. While they usually present a non-lethal threat to larger individuals, gnathiid parasitism of newly recruited juveniles from fishes with pelagic larvae can incur significant costs to recruits’ aerobic, competitive, escape response and swimming performance, as well as survival [10–15]. Many fishes engage in parasite-removal services [16], but these ‘cleaners’ tend to prefer larger clients [17]. Nonetheless, the presence of cleaner fishes can influence juvenile fish settlement patterns [18], further suggesting that the risk posed by parasitism-induced mortality is sufficiently important to shape eco-evolutionary patterns.

Parental care may protect juvenile fishes not only from predation, but also from the threat posed by gnathiids. The spiny chromis (*Acanthochromis polyacanthus*, herein ‘spiny chromis’) is the only coral reef fish on the Great Barrier Reef that has no pelagic larvae and exhibits parental care [1]. Parents guard benthic egg nests and protect juveniles from predators and conspecifics until they reach approximately 55 mm (standard length, SL) [19–21]. In the laboratory, the likelihood of mortality from one gnathiid (35%) does not differ between spiny chromis juveniles and other similar-sized damselfishes that do not exhibit parental care, such as the yellowtail demoiselle [12]. This suggests that spiny chromis juveniles are not inherently better adapted to a gnathiid attack than fish with a pelagic phase. Furthermore, as juveniles grow from 7 to 24 mm SL, laboratory experiments show that spiny chromis exposed to gnathiids switch from largely being hosts for gnathiids to being predators of gnathiids [22]. Importantly, if the larger fish had become parasitized by a gnathiid, they almost never ate the same engorged gnathiid afterwards, indicating that parasitism and predation are mutually exclusive [22]. This raises the question of whether parental care involves the consumption of parasites and functions as a defensive behaviour [23]. To investigate whether parental care confers protection to juvenile spiny chromis from gnathiid-induced mortality, we examine (i) whether the effect of parental presence affects the survival of juvenile spiny chromis exposed to gnathiids, and (ii) whether this form of protection is conferred through parents consuming the gnathiids. We hypothesize that in the presence of parental care, juvenile spiny chromis experience a reduction in gnathiid-induced mortality via parental consumption of gnathiid isopods.

## 2. Methods

### (a) Field observations of fish behaviour

Field observations of spiny chromis behaviour were done between December 2006 and January 2007 at Lizard Island, northern Great Barrier Reef, Australia (14.6645° S, 145.4651° E). Observations of fish with broods were used to identify the actively guarding parent for the survival experiment, confirmed upon capture as the male by the presence of a longer pointed genital papilla compared with a shorter round papilla in females [24]. For 34 observations, the percent of time spent guarding by adult category (both sexes, male, or female), and additional parental-care-associated behaviours were recorded (for methods, behaviours recorded and results, see electronic supplementary material, figure S1). A 5 min acclimation period was used prior to the start of the observation. The percentage of time spent guarding by adult category was then obtained by recording its occurrence once a minute for 15 min.

### (b) Survival experiment: fish survival

An aquarium experiment investigating how parent presence affected gnathiid-juvenile spiny chromis interactions was conducted between December 2006 and January 2007. An estimated 50 individuals from each brood and the actively guarding parent were collected on reefs surrounding Lizard Island. Broods were captured using two 25 × 20 × 35 cm fine soft approximately 1.5 mm mesh hand nets to minimize handling stress. Newly hatched (<10 d old) broods were selected based on fish size (approx. 5 to 9 mm SL, [25]). Broods were gently transferred to a quick-sealing bag (21 × 30 cm) with other individuals from the same brood. The size of brood individuals at capture was confirmed by holding a ruler next to the bag underwater. Individuals tended to vary little in size within a brood. The small size range in fish SL was selected because their probability of survival from a gnathiid infection is nearly 100% after 12 mm SL [11,22]. The parent was collected using a barrier (1 m × 2 m × 5 mm mesh) and hand (5 mm mesh) net and placed in a separate bag. The brood and parent were transferred to separate buckets with aerated seawater at the surface for transport to Lizard Island Research Station. Juveniles were not directly measured prior to experimentation due to their fragility and to avoid excessive stress.

**Figure 1.**
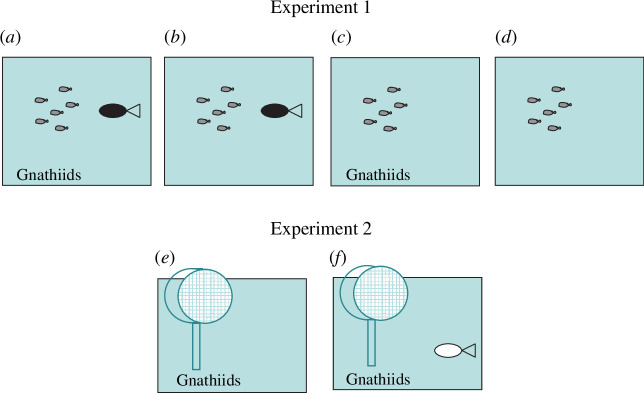
Schematic of treatments testing spiny chromis brood (grey fish symbols) survival (survival experiment) and spiny chromis adult (non-parent: white fish symbol) feeding rate on gnathiid isopods (feeding experiment). (*a*) parent (black fish symbol) and gnathiids, (*b*) parent and no gnathiid isopods, (*c*) no parent and gnathiids, (*d*) no parent and no gnathiids, (*e*) no adult and gnathiids, (*f*) adult and gnathiids. Disc mesh fitting and fish shelter for the survival experiment are not shown. Figure not to scale. See §2 and electronic supplementary material for more details.

Fish were placed in clear 50 cm width × 30 cm height × 30 cm diameter aquaria with a disc-shaped fine mesh fitting (30 mm width × 90 mm diameter polyvinyl chloride pipe with a 100 µm mesh on each end; for illustration of fitting, see figure 1) covering the outflow to prevent escape by the juveniles and gnathiids (figure 1*a*–*d*). Each aquarium was supplied with constant flowing seawater, an air-stone and a polyvinyl chloride pipe (10 cm length × 9 cm diameter) as a fish shelter. See electronic supplementary material for more detailed methods.

Fish were allowed to acclimate overnight before being used in the experiment and were fed daily in the morning with a diet of fish protein food pellets and freshly hatched live *Artemia* nauplii. The experiment comprised four treatments: (i) brood, parent and 50 gnathiids (*n* = 10), (ii) brood, parent and no gnathiids (*n* = 10); (iii) brood, no parent and 50 gnathiids (*n* = 13), (iv) brood, no parent, no gnathiids (*n* = 10) (figure 1*a–d*). Gnathiids were sourced from a gnathiid culture (for details on methods and culture, see electronic supplementary material). Fifty (except once *n* = 52) gnathiid juveniles with no engorged gut were added to the aquaria around 10.30 h. Aquaria and the order of treatments were allocated haphazardly as fish and gnathiids became available.

The number of surviving juveniles was recorded each following morning (approx. 10.30 h) over 3 days; juveniles which had died were removed. Detached engorged gnathiids were counted and removed each morning and in the afternoon (approx. 17.30 h); their counts were summed over the 3 days. At the conclusion of each 3-day trial, surviving fish were removed, and the aquarium water was passed through a sieve (62 µm) to recover and count the remaining gnathiids. The SL of the adult fish was measured. Adults and surviving juveniles were returned to their place of capture the following day.

Observations of fish and gnathiids during some trials were recorded to determine whether juveniles were at risk of becoming parasitized with a gnathiid and how juveniles and adults interacted during the trials (for methods, details on individual’s infection rates, behaviours recorded and results, see electronic supplementary material, figure S2, table S1). Four 15 min observations per trial were done; they were limited to two haphazardly selected trials per treatment combination due to time constraints.

### (c) Gnathiids in faeces of wild fish

To determine whether adult and juvenile spiny chromis eat gnathiids in the wild, we collected their faeces after 24 h in captivity. Collections of adult fish were conducted in November 2018 and January 2019. Broods and a parent (maximum five juveniles per brood) were only sampled in the November 2018 sampling trip (*n* = 14 adult-brood pairs), when young broods were abundant, but not in January 2019 as none were found at our sampling sites; for fish sample sizes (*n* = 8 to 23 per species per sampling trip) and fish sizes see electronic supplementary material, table S2. Adult fish or broods were placed separately in 10 l buckets with aeration overnight (24 h), then removed and measured; the contents of the bucket were filtered with a sieve (100 µm) to collect their faeces. To determine whether consuming gnathiids also occurs in a similar species that does not engage in parental care, we collected adult staghorn damselfish *Amblyglyphidodon curacao* (Pomacentridae) from the surrounding area, because they are similar in size and co-occur in the same habitat as spiny chromis, i.e. they occur above the reef in the water column, and feed on plankton and algae [26,27]. Partially digested gnathiids in faeces, easily identified by the undigested heads, were quantified in a Petri dish under a dissecting microscope (20 to 40× magnification). They were easily differentiated from parasitic gnathiids that had dropped off fish, which were intact [28].

### (d) Feeding experiment

An experiment investigating whether adult spiny chromis consumed gnathiids was conducted in February 2019. Specifically, we tested the persistence of gnathiids after being exposed to a ‘non-parent’ adult or ‘no fish’ treatment. Adults either had broods with relatively large-sized juveniles (>20 SL mm) or no discernible brood at the time of capture, and hence all were considered here as ‘non-parent’. Fish were collected from the lagoon and transported in buckets of aerated seawater to the laboratory as above. Fish were placed in test aquaria to acclimate for 24 h during which they were fed commercial fish flakes. Six white opaque plastic aquaria (30 cm width × 40 cm height × 30 cm depth) were used, each with an outlet pipe that had a fine disc mesh fitting attached, running seawater and an air-stone. Treatments (*n* = 16 per treatment) were allocated to aquaria haphazardly as fish and gnathiids became available (figure 1*e–f*). Once the trials began, the aquaria were covered with sunshade cloth to minimize disturbance to fish.

After fish acclimation, cultured gnathiids were collected as above in the morning. Twenty gnathiids were added to each aquarium (‘non-parent’ and ‘no fish’, figure 1*e–f*). After 24 h, the fish was removed and its SL measured in a plastic bag. The fish was then transferred to a holding aquarium and returned to its place of capture the following day. The seawater in the aquarium was filtered using a 62 µm sieve to collect the gnathiids. Gnathiids were counted using a Bogorov tray under a dissection microscope (20 to 40×).

## 3. Statistical analyses

### (a) Focal field observations of parent guarding behaviour

The percent time a single parent spent guarding the brood was tested using a Wilcoxon two-sample test (normal approximation) test with JMP Pro version 17 (SAS Institute Inc).

### (b) Survival experiment: fish survival

Analyses were conducted using R version 4.0.2 [29], unless otherwise specified. To test whether survival differed according to treatment, we first fit survival (yes/no) curves per parent and gnathiid presence/absence treatment combination with the package ‘coxme’ [30], using a model with parent and gnathiid presence/absence treatments as fixed effects and brood identity as a random effect. A full model was fitted using the function ‘coxph’. We tested the Cox model assumption of proportionality using the global test statistic using the functions ‘coxph’ and ‘cox.zph’. It was not significant, indicating the assumption was met (*χ*^2^ = 1.4212, d.f. = 3, *p* = 0.70). Type II Wald chi-squared tests were used to test for a significant difference among treatments using the function ‘Anova’ in package ‘car’ [31]. Whether parent SL differed between parented gnathiid and parented no gnathiids treatments was tested with an analysis of variance (ANOVA); one SL measure per treatment was not done. Parent SL did not differ between gnathiid treatments (no gnathiids: mean 80.8 mm ± 2.0 s.e.; gnathiids present: 83.3 ± 2.0; ANOVA: *F* = 0.8388; d.f. = 1, 16; *p* = 0.3733; electronic supplementary material, table S3).

### (c) Gnathiids in faeces of wild fish

Whether the proportions of adult fish with gnathiids present/absent in faeces differed between the two fish species was tested using a Fisher’s exact test, using JMP Pro version 17 (SAS Institute Inc). Only data from adults from November 2018 were analysed, because no gnathiids were found in the faeces of juveniles then, and none in the adults in January 2019.

### (d) Feeding experiment

To test for a difference in the number of gnathiids that had persisted after 24 h, between ‘non-parent’ adult or ‘no fish’ treatments, a generalized linear model (GLM) with treatment as a fixed categorical effect was conducted. We tested models with a Poisson and a Gaussian distribution; the former was selected using the Akaike information criterion. We used the package ‘stats’ and function ‘glm’ [29]. Residual diagnostic tests were done using the package ‘DHARMa’ [31]. Type II Wald chi-squared tests were used to test for a significant effect of fish treatment using the function ‘Anova’ in package ‘car’ [32].

## 4. Results

### (a) Focal field observations of parent guarding behaviour

The percent time a single parent guarded the brood differed between sexes (Wilcoxon two-sample test: *Z* = 6.667, *p *< 0.0001), with the male guarding the most (median 83%; 42/100, 25th/75th quantiles) and the female rarely (median 0%; 0/7, 25th/75th quantiles); the male–female parent pair guarded a median 17% (0/52, 25th/75th quantiles) of the time.

### (b) Survival experiment

**Figure 2.**
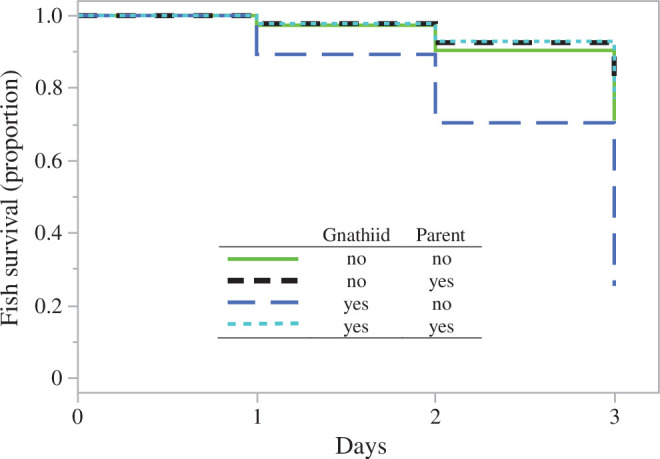
Kaplan–Meier survival curves for juvenile spiny chromis according to gnathiid isopod and parent presence treatments over number of days of exposure.

There was a significant interaction between gnathiid exposure and parent presence on fish survival over 3 days (Coxme, *χ*^2^ = 10.1, d.f. = 1, *p *< 0.0015; electronic supplementary material, table S4). A Kaplan–Meier plot was used to interpret the significant interaction (figure 2). This indicated a three times lower survival in fish exposed to gnathiids and held in the absence of a parent after 3 days (yes gnathiid–no parent: 25% mean survival), relative to the other three treatments, which were very similar to each other (no gnathiid–no parent: 71%, yes gnathiid–yes parent: 77%, no gnathiid–yes parent: 78%).

Across the 3 days of the survival experiment, in two parented trials, a total of 1 and 22 engorged gnathiids trial^−1^ were found on the bottom of the aquaria. In the four unparented trials, a total of 12, 2, 6 and 3 engorged gnathiids trial^−1^ were found on the bottom. Gnathiids without an engorged gut were occasionally observed rapidly swimming towards the parent.

During focal laboratory observations of adults, broods and gnathiids, some juveniles had a gnathiid attached (electronic supplementary material, table S1). The juveniles were often observed schooling with the rest of the brood. The brood was often moved by the parent from one area of the aquarium to the other. No infections were observed on a parent. For each of the broods held with a parent, some individuals were observed glancing off the parent. 'Glancing' involves the juvenile rapidly contacting the body of the parent with a forward thrust and the ingestion of mucus from their flanks [19]. These observations support our assumption that juveniles were at risk of being parasitized by gnathiids during our experiment and that adults and juveniles behaved in a relatively normal way compared with observations made in the wild (electronic supplementary material, figure S1).

### (c) Gnathiids in faeces of wild fish

Gnathiids were found in the faeces of adult spiny chromis and staghorn damselfish in November 2018 (abundance range, *n* of fish: 0 to 5, *n* = 23; 0 to 4, *n* = 12, respectively; electronic supplementary material, figure S3). The proportion of gnathiids present in faeces in November 2018 did not differ between species (spiny chromis: 17%, staghorn: 33%; percent with gnathiids; Fisher’s exact test, *p* = 0.4024). No gnathiids were found in either species in January 2019 (*n* = 13, *n* = 8, respectively). No gnathiids were found in spiny chromis juvenile faeces in November 2018.

### (d) Feeding experiment

**Figure 3.**
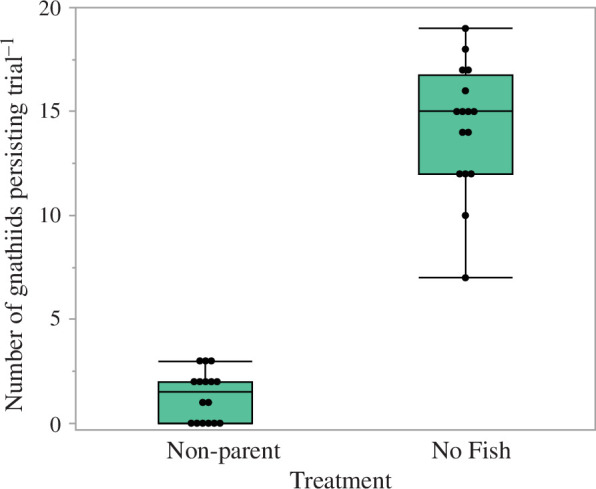
Number of gnathiids (out of initial 20) that persisted per trial after 24 h exposure to an adult non-parent spiny chromis or no fish treatment. Boxplots: centre line = median, box = interquartile, error bars = 90th and 10th percentiles and circles = outliers.

Of the 20 gnathiids added to an aquarium, the number that persisted after 24 h differed according to adult fish presence treatment (GLM: likelihood ratio test, *χ*^2^ = 201.15, d.f. = 1, *p *< 0.0001). This was due to 7.5% (median) of the 20 gnathiids persisting in the presence of a parent compared with 75% in the absence of a parent (figure 3). Thus, compared with an aquarium with no fish present, only 9% of gnathiids added to an aquarium persisted after 24 h when they were exposed to an adult non-parent spiny chromis.

## 5. Discussion

Without adult protection, the survival of juvenile spiny chromis in the wild is very low [21,33]. Here, we show that adult spiny chromis protect their offspring from ectoparasite-induced mortality. We found that a group of juveniles from the same brood held with a parent suffered lower rates of parasite-induced mortality than juveniles in trials without a parent. Analysis of the faeces of adult spiny chromis collected in the wild indicated they had consumed gnathiids, which was confirmed in a laboratory experiment. This suggests the mechanism of parental protection from gnathiids involves the consumption of those parasites by the parent. Morbidity and mortality in juvenile fishes from gnathiids can be severe [10–15]. These results highlight the potential hidden cost that gnathiid-induced mortality has on juvenile fish mortality, and consequently fish community structure, in marine ecosystems.

In the laboratory, the survival of juveniles that were exposed to gnathiids, and lacking a parent for 3 days, was three times lower (25%, average survival rate), relative to the other three treatments (71% to 78% per treatment). The small size of juveniles (approx. 5 to 9 mm SL) would have made them more likely to die from a gnathiid blood feeding, because gnathiid-related mortality in spiny chromis, and many other species from the same family (Pomacentridae), is associated with sizes <12 mm SL [11,12]. This mortality is most likely due to the proportionately high loss of fish blood volume per gnathiid blood meal [10–12]. Based on the mean engorged gut volume of first, second and third gnathiid juvenile stages [34], for small spiny chromis (10.7 mm SL, 0.038 g, 1.482 mm^3^ blood volume [35]) the percent blood volume removed per gnathiid juvenile stage is 7, 17 and 151% fish^−1^, respectively. Our experiment involved all stages, so feeding by any gnathiid is likely to have been detrimental to the survival of juvenile spiny chromis.

In the survival experiment, the starting ratio of gnathiids to juvenile abundance was 50 : 50 or 1 : 1. In the wild at our sites, the rate of gnathiids emerging from the reef in search of hosts is highly variable (averaging 0.5 to 34 gnathiids per m^2^ 12 h^−1^, depending on sampling time), although higher abundances up to 468 gnathiids per m^2^ 12 h^−1^ are common [36], suggesting that our working ratio of 1 : 1 is realistic. In the care of parents in the wild, the prevalence of gnathiids on juveniles <10 mm SL is 6% [22]. However, gnathiids may attach for as little as 3 h [10]. Thus, the cumulative effects of gnathiids within the first few days after hatching are expected to be larger than that predicted by the prevalence of observed juveniles with attached gnathiids because more fish could be parasitized over time, even repeatedly. Mortality of coral reef fishes during settlement is extremely high and is largely attributed to predation. A meta-analysis estimated an average mortality rate of 30% per day, but it can be as high as 60%, for newly settled juvenile fishes [37,38]. However, gnathiids not only can kill juveniles, they also have sublethal effects on juveniles including increased fish stress levels, as well as reduced swimming speed, escape responses, settlement success and competitive success, all of which likely predispose fish to mortality [10–15]. While predation is undoubtedly an important selective pressure, our study indicates that ectoparasite-induced mortality may also be an important and currently underestimated mortality source for juvenile fishes settling on the reef.

In November 2018, gnathiids were present in the faeces of wild-caught adult spiny chromis (17%) and in another, ecologically similar, control species that does not exhibit parental care, the staghorn damselfish (33%). This did not occur in January 2019. There is no evidence of seasonality in the free-living stages of gnathiids that would explain the absence of gnathiids in the faeces in January 2019, though they can vary greatly temporally and spatially [36]. That the proportions in the faeces in November 2018 did not differ significantly between fish species suggests that feeding on gnathiids is not a behaviour unique to spiny chromis. Indeed, other than some cleaner fishes*,* which feed almost exclusively on the parasitic stage of gnathiids, non-cleaner fishes also have gnathiids present in their faeces with planktivorous damselfishes having the most gnathiids across non-cleaners from different functional feeding groups [28,39,40]. Given the planktivorous diet of these damselfishes, it is likely gnathiids were consumed while free-living in the water column, and not when attached to a host [26,41]. That adult spiny chromis can eat many gnathiids was confirmed in the laboratory; only 9% of gnathiids added to an aquarium persisted after 24 h when held with an adult, compared with the treatment lacking a fish. Whether ingestion of gnathiids is targeted or incidental remains unclear. No gnathiids were found in faeces of wild juvenile spiny chromis. This was unsurprising given their small size, and agrees with previous laboratory feeding trials [22]. This indicates the juvenile fish tested in the survival experiment were also likely too small to eat the gnathiid before it parasitized the fish, despite being at high risk of parasitism by gnathiids.

Consumption differs from other (causal or not) fish defences against gnathiid parasitism, including cleaning behaviour, mucous cocoons and adult nocturnal and larval pelagic migrations [11,16,42–44]. Additionally, some organisms can act as an alternate host that inadvertently distract free-living parasites away from other hosts [45]. Here, the larger spiny chromis adults provided a greater alternate target area to juvenile fish for gnathiids to encounter. However, instead of the alternative (i.e. the larger parent) becoming the host, it consumed the parasite. Indeed, where parents and gnathiids were present, gnathiids were occasionally observed attempting to parasitize the parent. Predation on parasites is widespread in nature and it can significantly affect their populations and food webs [46,47]. Other potentially protective behaviours we observed in the juveniles, such as ‘glancing’ [19] and grouping behaviours [48], were not effective here. Juvenile survival, in all treatments other than when gnathiids were present without parents, was similar, regardless of gnathiid exposure.

That spiny chromis exhibit parental care is inextricably linked to their lack of a pelagic larval stage. In species with pelagic larvae, mortality declines exponentially with increasing size at settlement [37]. However, spiny chromis juveniles are relatively small compared with settlement-stage coral reef fishes, suggesting that the benefits of parental care, at least as it pertains to gnathiid-induced fitness costs, are comparatively large at this time. Thus, protection from this important infectious mortality source may be a contributing selective factor for this life-history strategy. Our observed reduction in mortality from gnathiids in spiny chromis juveniles <12 mm SL, when under the care of a parent, suggests that parental care enables very small individuals to bypass the pelagic larval phase, a phase hypothesized to allow other fishes to avoid and outgrow some reef-based ectoparasites [11,49]. In the wild, juveniles were guarded mostly by the male (83%, median time). Unbalanced guarding between the spiny chromis parents has been noted before, but has not been sexually differentiated [33]. Whether the other reef fishes that care for broods (i.e. *Altrichthys* spp. [50]) may also benefit from parental defence against parasites remains to be tested.

## 6. Conclusions

Overall, our results highlight that parental care substantially reduces gnathiid-induced mortality in spiny chromis broods. Parents did so by eating these ectoparasites, irrespective of whether consumption involved an incidental foraging behaviour or an adaptation involving targeted consumption of gnathiids to prevent parasitism of their young. Furthermore, in addition to causing mortality, gnathiids have sublethal effects on other juvenile damselfishes including increased stress levels, and reduced swimming speed, escape responses, settlement success and contest success over shelter sites [10,13,14]. These may lead to increased vulnerability to predation. Taken together, this work emphasizes that mortality due to gnathiid infection may be under-appreciated as a selective agent across marine systems.

## Data Availability

The datasets supporting this article are at Figshare [51]. Supplementary material is available online [52].
